# Interaction of HLA Class II rs9272219 and *TMPO* rs17028450 (Arg690Cys) Variants Affects Neuromyelitis Optica Spectrum Disorder Susceptibility in an Admixed Mexican Population

**DOI:** 10.3389/fgene.2021.647343

**Published:** 2021-07-15

**Authors:** Sandra Rosas-Madrigal, María Teresa Villarreal-Molina, José Flores-Rivera, Verónica Rivas-Alonso, Luis Rodrigo Macias-Kauffer, Graciela Ordoñez, María del Carmen Chima-Galán, Víctor Acuña-Alonzo, Gastón Macín-Pérez, Rodrigo Barquera, Julio Granados, Ricardo Valle-Rios, Teresa Corona, Alessandra Carnevale, Sandra Romero-Hidalgo

**Affiliations:** ^1^Laboratorio de Enfermedades Mendelianas, Instituto Nacional de Medicina Genómica, Mexico City, Mexico; ^2^Laboratorio de Genómica de Enfermedades Cardiovasculares, Instituto Nacional de Medicina Genómica, Mexico City, Mexico; ^3^Laboratorio Clínico de Enfermedades Neurodegenerativas, Instituto Nacional de Neurología y Neurocirugía “Manuel Velasco Suarez” (INNN), Mexico City, Mexico; ^4^Unidad de Genómica de Poblaciones Aplicada a La Salud, Facultad de Química, UNAM/INMEGEN, Mexico City, Mexico; ^5^Laboratorio de Neuroinmunología, INNN, Mexico City, Mexico; ^6^División de Medicina Genómica, Centro Médico Nacional “20 de Noviembre”, ISSSTE, Mexico City, Mexico; ^7^Escuela Nacional de Antropología e Historia, Mexico City, Mexico; ^8^Department of Archaeogenetics, Max Planck Institute for the Science of Human History, Jena, Germany; ^9^Departamento de Trasplantes, Instituto Nacional de Ciencias Medicas y Nutrición “Salvador Zubirán”, Mexico City, Mexico; ^10^División de Investigación, Facultad de Medicina, Unidad de Investigación en Inmunología y Proteómica, Hospital Infantil de México Federico Gómez, Universidad Nacional Autónoma de México, Mexico City, Mexico; ^11^Departamento de Genómica Computacional, Instituto Nacional de Medicina Genómica (INMEGEN), Mexico City, Mexico

**Keywords:** NMOSD, *TMPO*, HLA, genetic interaction, rs17028450, rs9272219

## Abstract

Neuromyelitis Optica Spectrum Disorder (NMOSD) is a demyelinating autoimmune disease of the central nervous system, more prevalent in individuals of non-European ancestry. Few studies have analyzed genetic risk factors in NMOSD, and HLA class II gene variation has been associated NMOSD risk in various populations including Mexicans. Thymopoietin (*TMPO*) has not been tested as a candidate gene for NMOSD or other autoimmune disease, however, experimental evidence suggests this gene may be involved in negative selection of autoreactive T cells and autoimmunity. We thus investigated whether the missense *TMPO* variant rs17028450 (Arg630Cys, frequent in Latin America) is associated with NMOSD, and whether this variant shows an interaction with HLA-class II rs9272219, previously associated with NMOSD risk. A total of 119 Mexican NMOSD patients, 1208 controls and 357 Native Mexican individuals were included. The HLA rs9272219 “T” risk allele frequency ranged from 21 to 68%, while the rs17028450 “T” minor allele frequency was as high as 18% in Native Mexican groups. Both rs9272219 and rs17028450 were significantly associated with NMOSD risk under additive models (*OR* = 2.48; *p* = 8 × 10^–10^ and *OR* = 1.59; *p* = 0.0075, respectively), and a significant interaction between both variants was identified with logistic regression models (*p* = 0.048). Individuals bearing both risk alleles had an estimated 3.9-fold increased risk of NMOSD. To our knowledge, this is the first study reporting an association of *TMPO* gene variation with an autoimmune disorder and the interaction of specific susceptibility gene variants, that may contribute to the genetic architecture of NMOSD in admixed Latin American populations.

## Introduction

As other autoimmune diseases, Neuromyelitis Optica Spectrum Disorder (NMOSD) is a multifactorial disorder that results from complex interactions between genetic and environmental factors. Its worldwide prevalence has been estimated to range between ∼0.5 and 4/100,000 and may be up to 10/100,000 depending on geographical location and ethnicity. It has been consistently suggested that NMOSD is more frequent in non-European populations. East Asians and populations with African ancestry have reported a higher prevalence of this disease (Hor et al., 2020).

In Latin America, admixed populations with lower proportions of European ancestry have higher relative frequencies of NMOSD (Alvarenga et al., 2017). We recently proposed that Native American ancestry contributes to NMOSD susceptibility, as Mexican NMOSD patients showed significantly higher proportions of Native American ancestry than controls. In addition, we performed a genome-wide association study (GWAS) identifying a NMOSD risk genetic variant within HLA class II region (rs9272219) that is in linkage disequilibrium with HLA DRB1^∗^16:02, previously associated with NMOSD in Southern Han Chinese, Japanese and Brazilian populations (Wang et al., 2011; Yoshimura et al., 2013; Kay et al., 2019; Romero-Hidalgo et al., 2020). Other HLA class II risk alleles have been associated with NMOSD in different ethnic groups. A limited number of candidate gene association studies have also reported associations of NMOSD with individual polymorphisms in a few genes involved in immune function, including PD-1, IL-17, IL-7R, CD226, and CD58 (Asgari et al., 2012; Liu et al., 2012; Wang et al., 2012; Kim et al., 2014; Zhuang et al., 2015).

Thymopoietin (*TMPO*) has not been previously explored as a candidate gene for NMSOD or other autoimmune disease. *TMPO* is located on chromosome 12q22, is highly expressed in immune cells (BioGP, 2020) and plays an important role in T cell differentiation (Basch and Goldstein, 1974; Silva et al., 2009). It encodes thymopoietin or LAP2 known to be involved in nuclear architecture and chromatin organization by interacting with structural nuclear proteins such as lamins (Lunin et al., 2020). LAP2 binds to lamin B (Foisner and Gerace, 1993) and regulates the correct distribution of lamin B in the nucleus through its carboxy-terminal domain (Brady et al., 2018). On the other hand, lamin B plays a role in maintaining cortical and medullar compartments of the thymus by inducing gene expression in Thymic Epithelial Cells (TECs) (Yue et al., 2019). Importantly, altered lamin B function may induce the generation of ectopic transcripts encoding Peripheral-Tissue Antigens (PTAs) by influencing the Autoimmune Regulator (AIRE), leading to autoimmunity (Abramson et al., 2010). Thus, genetic variants affecting interaction of LAP2 and lamin B may indirectly affect the normal function of AIRE in the thymus.

Rs17028450 is a missense polymorphism (Arg690Cys) in the *TMPO* gene, and the 690 Cys protein was found to decrease the interaction LAP2 and lamin A proteins *in vitro* (Taylor et al., 2005). Interestingly, the derived allele is most frequent in Latin American populations (14%), and less common in Asians, Africans and Europeans (<2%). Thus, we aimed to evaluate whether the *TMPO* rs17028450 missense variant is associated with NMOSD, and whether this variant interacts with HLA class II variation affecting NMOSD susceptibility in the admixed Mexican population.

## Materials and Methods

### Study Population

Microarray data of 119 previously described admixed Mexican NMOSD patients (79% female) and 1,208 controls (61% female) (Romero-Hidalgo et al., 2020) were used for the analyses. In addition, *TMPO* rs17028450 genotypes were determined using Taqman probes in 85 systemic lupus erythematosus (SLE) patients recruited from the Instituto Nacional de Ciencias Médicas y Nutrición “Salvador Zubirán,” and 104 multiple sclerosis (MS) patients recruited from the DNA bank of the Genomic Medicine Division at the Centro Médico Nacional 20 de Noviembre del Instituto de Seguridad y Servicios Sociales de los Trabajadores del Estado (ISSSTE). SLE patients were diagnosed according to the 1982 SLE classification criteria (Watanabe et al., 1985) and MS was diagnosed according to McDonald criteria (Sadaka et al., 2012). In addition, TMPO genotypes from 3 different Native Mexican groups were analyzed: 53 previously described Nahuas and 19 Totonacs (Romero-Hidalgo et al., 2017) using Taqman probes, and 82 Mayans with type 2 diabetes recruited from rural health centers in the State of Yucatan with available exome data. Finally, allele frequencies of HLA rs9272219 were evaluated in 138 Nahuas, 24 Totonacs, 45 Zapotecs and 68 Mayas using available microarray data (Romero-Hidalgo et al., 2017). Protocols for each cohort were approved by their respective Institutional Ethics and Research Committees (No. 66/14, No. DIC/491/14).

### Genotyping

Genomic DNA was extracted from blood samples with the QIAamp DNA Blood Midi/Maxi kit (Quiagen) according to the supplier’s instructions. DNA integrity, purity and concentrations were determined by NanoDrop^®^ spectrophotometry (NanoDrop One/One^*c*^ Thermo scientific). The *TMPO* rs17028450 single nucleotide polymorphism (SNP) was genotyped by Taqman^®^ assays (Life Technologies Company) using a viia^TM^ seven real-time PCR instrument. Genotype were assigned automatically by measuring allele specific fluorescence using TaqMan^®^ Genotyper^TM^ Software (Applied Biosystems). PCR mix included 15 ng of genomic DNA, 0.45 μM of oligonucleotide (VIC/FAM AGAAGTATGCAAAGTAATTAAAAAG[C/T]GTGGAAATAA ACACTAGTAAAATTA), 2.5 μl of TaqMan master mix (Applied Biosystems, Foster city, CA, United States) and ddH_2_O for a final volume of 5 μl per reaction. The amplification protocol was as follows: one cycle of 10 min at 95°C (denaturing), followed by 40 cycles of 15 s at 95°C (denaturing), and 1 min at 60°C (annealing) and 1 min at 72°C (extension). *TMPO* rs17028450 genotypes from a subset of 19 NMOSD samples were validated by Sanger sequencing according to the standard protocol used at the High Technology Core of National Institute of Genomic Medicine (HTC_INMEGEN). Primer sequences were: Forward 5′-tcagcagttggacttagcactc and Reverse 5′-tgtcctaggtataaaggaggatgc. No discordant genotypes were found.

### Statistical Analyses

Logistic regression models were used to test associations of NMOSD with single SNPs under additive and dominant inheritance models, and to test gene-gene interactions adding a multiplicative interaction term in the corresponding logistic regression model. Finally, a genetic risk score (GRS) was tested by summing the “T” risk alleles of HLA rs9272219 and *TMPO* rs17028450 variants (0, 1, 2, 3, or 4 risk alleles). A weighted GRS was also calculated as the sum of the effect estimates from a logistic regression analysis with an additive inheritance model, multiplied by the number of risk alleles (Igo et al., 2019). All logistic regression models were adjusted for sex and two principal components as covariates. The principal component was obtained from Romero-Hidalgo et al. (2020). Because age was not available for all NMOSD cases, it was not included as covariate in the analyses. An independent *t*-test was used to compare mean GRS values between cases and controls. Statistical analyses were performed using R software environment (R-Software, 2021). The Geography of Genetic Variants (GGV) were used to map the geographic distribution of the genetic variants (Marcus and November, 2017).

## Results

**FIGURE 1:**
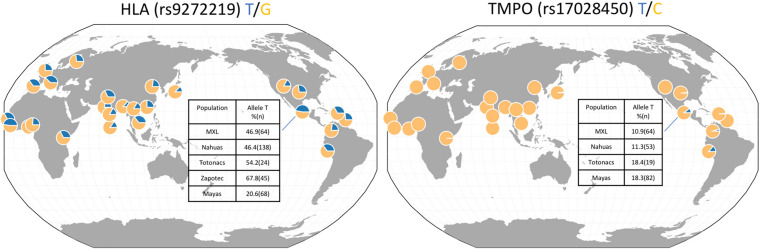
HLA class II rs9272219 and *TMPO* rs17028450 allele frequencies in worldwide populations and in Native Mexican groups.

Figure 1 shows the worldwide derived allele frequencies of HLA class II rs9272219 and *TMPO* rs17028450 based on populations included in 1,000 genomes project. Clearly, *TMPO* rs17028450 is an ancestry-specific polymorphism almost private to the Americas. The frequency of the derived “T” allele is lower than 2% in Asian, African and European populations, and 10.9 and 14.1% in Mexican American and Peruvian populations, respectively. In contrast, the HLA rs9272219 “T” allele is frequent in all populations, ranging from 13.4% in South and East Asian populations to 47% in Sierra Leone and Mexican Americans. In Native Mexican populations, the HLA “T” risk allele ranged from 20.6% in Mayans to 67.8% in the Zapotec population, whereas, the *TMPO* derived allele showed a more homogenous allele frequency among Native Mexican populations (11–18%).

For the case-control association study, a total of 119 NMOSD patients (79% female) and 1,208 apparently healthy controls (61% female) were included in the analysis. As shown in Table 1, HLA rs9272219 and *TMPO* rs17028450 risk allele frequencies were higher in NMOSD patients (67.2 and 23.5%, respectively) as compared to controls (41 and 13.5%, respectively). Additive and dominant inheritance models were evaluated for both genetic variants and all showed significant associations in the admixed Mexican population. The HLA “T” allele showed significant association with NMOSD risk (*OR* = 2.48; *p* = 8 × 10^–10^ and *OR* = 2.95; *p* = 0.0001, additive and dominant models, respectively). The *TMPO* “T” allele was also significantly associated with NMOSD risk (*OR* = 1.59; *p* = 0.0075 and *OR* = 1.88; *p* = 0.002, additive and dominant models, respectively).

**TABLE 1:** Association of HLA rs9272219 and *TMPO* rs17028450 with NMOSD in the Mexican Population.

	**Chr**	**RA**	**RAF NMOSD (*n* = 119)**	**RAF Ctrl (*n* = 1208)**	**Inheritance model**	**OR (95% CI)**	***p***
rs9272219	6	T	67.2%	41.0%	Additive	2.48 (1.86–3.34)	8 × 10^–10^
					Dominant	2.95 (1.75–5.29)	0.0001
rs17028450	12	T	23.5%	13.5%	Additive	1.59 (1.13–2.23)	0.0075
					Dominant	1.88 (1.25–2.80)	0.002

*RA, Risk allele; RAF, Risk allele frequency; OR, Odds ratio; CI, Confidence intervals.*

*p, p-values were obtained using logistic regression analysis adjusted for sex and two principal components.*

Because *TMPO* rs17028450 was found to be associated with NMOSD, we explored the allele frequencies of this variant in patients with other autoimmune diseases. The “T” allele frequency was 12.2% in a group of 104 MS patients, and 15.8% in a group of 85 SLE patients, however allele frequencies were not significantly different as compared to controls (13.5%).

**FIGURE 2:**
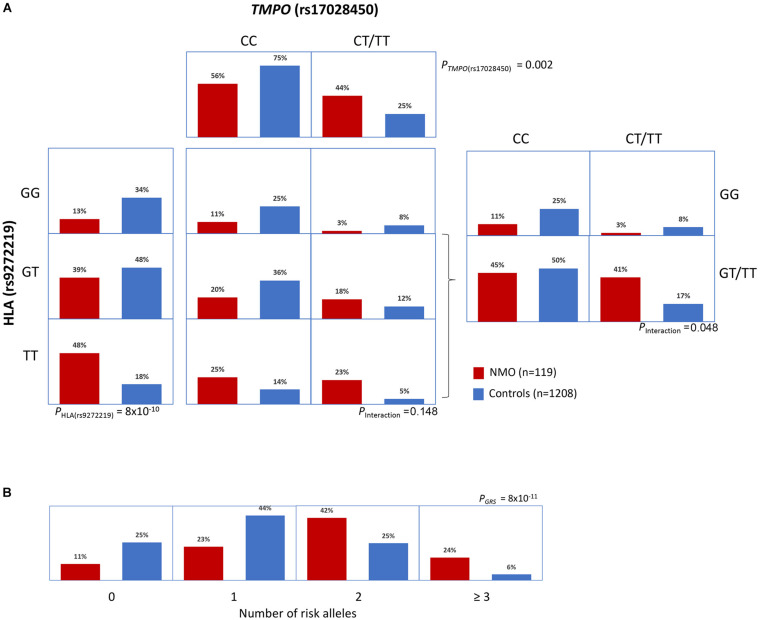
Interaction and genetic risk score analyses. **(A)** Distribution of NMOSD cases and controls across genotypes for HLA rs9272219 and *TMPO* rs17028450 genetic variants. The *TMPO* variant was analyzed assuming a dominant inheritance model mainly because of the reduced number of *TMPO* “TT” homozygous individuals. **(B)** Distribution of cumulative risk alleles (HLA rs9272219 and *TMPO* rs17028450) in cases and controls.

In order to assess a possible HLA-*TMPO* gene interaction we compared HLA risk variant frequencies in NMOSD cases and controls, stratified by the absence or presence of the *TMPO* risk allele (“CC” vs. “CT/TT” genotypes) (Figure 2A). Interestingly, the distribution of cases and controls across the three HLA genotypes (GG, GT, TT) differed according to the presence or absence of the *TMPO* risk allele. We observed a case-control ratio of 2.7 (0.48/0.18) in individuals with HLA “TT” genotype without considering *TMPO* genotypes. However, when stratified by the absence or presence of the TMPO risk allele, case control ratios in HLA “TT” genotypes were 1.8 (0.25/0.14) in the absence, and 4.6 (0.23/0.05) in the presence of the TMPO “T” risk allele. Similarly, the case-control ratio in individuals bearing the *TMPO* “T” risk allele is 1.8 (0.44/0.25). However, when stratified by the absence or presence of the HLA “T” risk allele case-control ratios were 0.4 (0.3/0.8) and 2.41 (0.41/0.17), respectively. According to the logistic regression model, the interaction was significant when both SNPs are considered dominant (*p* = 0.048) and only show a tendency for the HLA (additive)-*TMPO* (dominant) scenario (*p* = 0.148; Table 2).

**TABLE 2:** HLA rs9272219 and *TMPO* rs17028450 in NMOSD, interactions and genetic risk score analyses.

**Model**	**Predictors**	**OR (95% CI)**	***p***
Interaction	rs9272219*_add_*	2.10 (1.47–3.06)	7.1 × 10^–5^
	rs17028450*_*d*__*om*_*	1.07 (0.42–2.57)	0.879
	rs9272219*_*a*__*dd*_* × rs17028450*_*d*__*om*_*	1.56 (0.86–2.88)	0.148
Interaction	rs9272219*_*d*__*om*_*	1.95 (1.07–3.81)	0.037
	rs17028450*_*d*__*om*_*	0.57 (0.12–1.85)	0.398
	rs9272219*_*d*__*om*_* × rs17028450*_*d*__*om*_*	3.90 (1.12–18.25)	0.048
Genetic risk score	0, 1, 2, ≥ 3 risk alleles	2.14 (1.71–2.71)	8.8 × 10^–11^

*Subscript add and dom refer to additive and dominant inheritance model, respectively. All models were adjusted for sex and two principal components. Only the dominant inheritance model was assessed for rs17028450 because of the low frequency of double homozygotes.*

We used a GRS to analyze the cumulative contribution of HLA and *TMPO* variants (Figure 2B). The mean GRS was significantly higher in cases (1.82 ± 0.97) than in controls (1.12 ± 0.86; *p* = 4.6 × 10^–12^) and showed a strong and significant association with NMOSD risk, for both the unweighted (*OR* = 2.14; *p* = 8.8 × 10^–11^) and weighted models (*OR* = 2.78; *p* = 2.5 × 10^–11^).

## Discussion

As other autoimmune diseases, NMOSD is a multifactorial disorder that results from complex interactions between genetic and environmental factors. First considered a variation of MS, it was identified as a distinct autoimmune entity after the discovery of positive antiaquaporin-4 antibodies in 2004. Although significant progress has been made in the clinical and epidemiological characterization of NMOSD, genetic and environmental risk factors involved in this disorder are still unclear, in part because of its low worldwide prevalence (Lennon et al., 2005; Paul et al., 2020).

Only four genome-wide association studies have been conducted in NMOSD, including a small number of cases per study (∼200). In spite of their low statistical power, three independent GWAS identified SNPs within the MHC region associated with NMOSD in individuals of European ancestry (Estrada et al., 2018), in Admixed Mexicans (Romero-Hidalgo et al., 2020) and in the Japanese population (Matsushita et al., 2020); while a GWAS in Koreans did not identify any variants associated with NMOSD susceptibility with genome-wide significance (Kim et al., 2010). The GWAS in the Mexican and Japanese populations reported similar results, as both identified a genome-wide significant association with rs1964995, although the lead SNP in the Mexican population was rs9272219 (Matsushita et al., 2020; Romero-Hidalgo et al., 2020). Moreover, both studies reported HLA DRB1^∗^16:02 and DRB1^∗^08:02 as NMOSD susceptibility alleles. It is noteworthy that rs9272219 risk allele frequencies in 3 of the Native Mexican populations (Nahuas, Totonacs, and Zapotecs) are among the highest reported worldwide. Interestingly, rs9272219 and rs1964995 have been associated with other autoimmune diseases such as rheumatoid arthritis and dermatomyositis in different populations (Eleftherohorinou et al., 2011; Hao et al., 2014).

Few non-HLA candidate gene studies have been conducted in NMOSD (Asgari et al., 2012; Liu et al., 2012; Wang et al., 2012; Kim et al., 2014; Zhuang et al., 2015), and as far as we know, *TMPO* has not been previously identified as a susceptibility gene in GWAS, nor has it has been studied as a candidate gene for any autoimmune disorder. Although experimental evidence on the role of *TMPO* and LAP2 in autoimmunity is limited, it has been demonstrated that LAP2 binds to lamin B1 *in vitro* (Foisner and Gerace, 1993). Furthermore, the carboxi-terminal domain of LAP2 is required to maintain the correct nuclear distribution of lamins A, B1 and B2 in cancer cells (Brady et al., 2018) suggesting that *TMPO* mutations may destabilize lamin distribution in certain cell types. This might be relevant since lamin B1 overexpression reduces the expression of the autoimmune regulator (AIRE), which in turn could inhibit the expression of a battery of peripheral-tissue self-antigens (PTAs), an essential for immunological tolerance by promoting the development of autoreactive T cells in thymic stromal cells (Abramson et al., 2010).

Notably, rs17028450 was first identified in a patient with dilated cardiomyopathy (DCM), and the LAP2 630Cys form of the protein was found to decrease the interaction of LAP2 with the lamin A terminus *in vitro* (Taylor et al., 2005). However, it is unknown whether this amino acid substitution affects LAP2 interaction with lamin B1, has other functional consequences in other specific cell types, or is involved in susceptibility to autoimmunity. The rs17028450 minor “T” allele is frequent in Latin Americans but rare in other continental populations (<2%), with frequencies as high as 18% in Native Mexican groups including Nahuas, Totonacs, and Mayans. To our knowledge, this is the first report associating *TMPO* gene variation with an autoimmune disease (NMOSD) using a candidate gene approach, under both additive (*OR* = 1.59, *p* = 0.0075) and dominant (*OR* = 1.88, *p* = 0.002) models.

Interaction effects are difficult to assess because the number of multilocus genotype combinations increases exponentially as additional SNPs are considered, and larger sample sizes are needed to estimate the corresponding effects. However, it is important to seek interactions since the effect of a genetic variant can be masked by the effect of other variants (VanderWeele, 2010). Because of this difficulty, and particularly for diseases of low prevalence, interactions have been sought using the candidate gene approach. Using this approach, we observed an interaction between HLA rs9272219 and *TMPO* rs17028450 with an estimated odds ratio of 3.9 for individuals bearing both risk alleles. To our knowledge, this is the first study reporting gene interactions affecting NMOSD risk. Epistatic interactions between HLA and non-HLA genetic variants have been previously reported for other autoimmune diseases including SLE, MS, rheumatoid arthritis, ankylosing spondylitis and psoriasis (Matzaraki et al., 2017; Diaz-Gallo et al., 2018; Hanson et al., 2020). In addition, a significant interaction between r9273363 (HLA DQB1) and SNPs rs9272219 (HLA DQA1) was reported in RA and type 1 diabetes, and this HLA region was identified as epigenetically active in B cells (Woo et al., 2017). Since Lamin B1 and LAP2 are expressed in the thymus, the LAP2 690Cys variant may indirectly affect AIRE function in medullary TECs, perhaps altering the presentation of AQP4 or other antigens in the thymus, allowing autoreactive T cells that recognize these antigens to escape negative selection. If this occurs in epigenetically active B cells expressing the HLA DQA1 rs9272219 “T” allele, this may favor the production of anti-AQP4 or other autoantibodies.

Some limitations of the study must be pointed out. Firstly, the association between *TMPO* rs17028450 with NMOSD requires confirmation in other populations, particularly in Latin Americans because of the higher frequency of this variant and the relatively higher prevalence of NMOSD. Secondly, although rs17028450 was not associated with MS or SLE (*p* > 0.26), it should be tested in larger cohorts of MS and SLE cases, and in other autoimmune diseases. Finally, further association and functional studies are required to confirm the interaction observed between HLA rs9272219 and *TMPO* rs17028450 and to elucidate how these variants may confer NMOSD susceptibility in combination. Although the sample size is small, our data support a model whereby the contribution of co-inherited risk alleles changes the individual’s predisposition to NMOSD, as probably occurs in many immune cell-mediated diseases.

## Data Availability Statement

The raw data for the NMO patients analyzed in the current study are available from the corresponding author on reasonable request. Access to the control dataset used in this manuscript was obtained through a formal request to the Consortium for the Analysis of the Diversity and Evolution of Latin America steering committee.

## Ethics Statement

The studies involving human participants were reviewed and approved by protocols for each cohort were approved by their respective Institutional Ethics Committees: Instituto Nacional de Medicina Gen mica, Instituto Nacional de Neurología y Neurocirugía “Manuel Velasco Suarez”, Instituto Nacional de Ciencias Medicas y Nutrición “Salvador Zubirán” and Centro Médico Nacional 20 de Noviembre del Instituto de Seguridad y Servicios Sociales de los Trabajadores del Estado. The patients/participants provided their written informed consent to participate in this study.

## Author Contributions

SR-M performed the genotyping. SR-M, AC, and SR-H conceived and designed study. SR-H and LM-K performed data analysis. TC, JF-R, and VR-A were responsible for recruiting NMOSD patients. GO and MC-G were responsible for providing multiple sclerosis patients samples. JG was responsible for providing systemic lupus erythematosus patients samples. VA-A, GM-P, and RB were responsible for providing Native Mexicans samples. RV-R contributed to analysis and interpretation of results. SR-M, MV-M, and SR-H wrote the manuscript. All authors contributed to the article and approved the submitted version.

## Conflict of Interest

The authors declare that the research was conducted in the absence of any commercial or financial relationships that could be construed as a potential conflict of interest.
